# Application of Rapid Rehabilitation Surgical Nursing Combined With Continuous Nursing in Self-Care Ability, Medication Compliance and Quality of Life of Renal Transplant Patients

**DOI:** 10.3389/fsurg.2022.844533

**Published:** 2022-02-21

**Authors:** Linyan Song, Qing Jin, Liying Zhu, Zhe Liu, Wenjuan Cheng

**Affiliations:** ^1^Nursing Department, Beijing Chao-Yang Hospital, Capital Medical University, Beijing, China; ^2^Department of Urology, Beijing Chao-Yang Hospital, Capital Medical University, Beijing, China

**Keywords:** kidney transplantation, rapid rehabilitation surgical nursing, continuing nursing, self-protection ability, quality of life

## Abstract

**Objective:**

To explore the effects of rapid rehabilitation surgery (FTS) nursing combined with continuous nursing on self-care ability, medication compliance and quality of life of patients after renal transplantation.

**Methods:**

Sixty patients who received kidney transplantation in our hospital from January 2019 to January 2021 were randomly divided into the control group and the observation group with 30 patients in each group according to the random number table method. The control group was given FTS nursing, while the observation group was given continuous nursing on the basis of the control group. General data were collected and compared between the two groups. Postoperative indexes such as the time of first intake and the like of patients in the two groups were recorded. The patients' comfort, self-care ability, medication compliance and quality of life after renal transplantation were evaluated in the two groups. During the follow-up, the hospitalization of patients with complications was recorded.

**Results:**

There was no significant difference in the first intake, blood glucose, creatinine, urea nitrogen, blood potassium or postoperative hospital stay between the two groups (*P* > 0.05). There was no significant difference in the postoperative physical, mental, psychological, social and environmental dimensions between the two groups (*P* > 0.05). The scores of cognitive symptom management, exercise and communication with doctors in the two groups in post-intervention were higher than those in pre-intervention, and the scores in the observation group in post-intervention were higher than those in the control group (*P* < 0.05). The medication compliance in the observation group (93.33%) was higher than that in the control group (70.00%) (χ^2^ = 5.455, *P* = 0.020). In post-intervention, the scores of quality of life of the observation group were higher than those of the control group (*P* < 0.05). The admission rate of complications in the observation group (10.00%) was lower than that in the control group (30.00%) (χ^2^ = 3.750, *P* = 0.035).

**Conclusion:**

FTS nursing can help renal transplantation patients to obtain more stable postoperative blood pressure, renal function and other indicators and comfort. On this basis, combined with continuous nursing can improve patients' self-care ability and medication compliance, which is of great significance to improve the quality of life of patients.

## Introduction

Kidney transplantation is an effective treatment for end-stage renal disease. In recent years, with the development of medical technology, the survival rate of grafts after kidney transplantation has obviously improved, which is conducive to prolonging the life span of patients and improving the quality of life (1). Good hemodialysis and peritoneal dialysis are the basis of kidney transplantation. Good perioperative preparation, safe anesthesia techniques and implementation of intraoperative heat preservation measures are the key to ensure the success of surgery, reduce postoperative complications, and promote the recovery of patients (2). Fast-track surgery (FTS) was first proposed by KehIet et al. (3) of Denmark, and its main purpose is to optimize the perioperative treatment plan through a series of optimization measures with evidence-based medical evidence, so as to relieve the pressure of patient's physical and mental trauma, and thus achieve the purpose of rapid recovery and reduce the total mortality. After the concept of FTS was introduced into China, it was widely used in many surgical fields such as orthopedics, cardiothoracic surgery, breast surgery, gastrointestinal surgery, etc. Its advantages are gradually recognized by the medical profession. Its main contents include perioperative nutritional support and liquid management, emphasizing oxygen supply, early feeding and minimally invasive surgery (4). FTS nursing requirements, in actual clinical application, according to the patients' age, type of surgery, operation time, fluid loss during operation and other factors, formulate specific personalized nursing scheme for patients (5). After kidney transplantation, the use of a large number of immunosuppressants leads to the decline of the body's resistance. At the same time, complications such as rejection, delayed recovery of transplanted renal function and urinary fistula bring difficulties to the nursing and patient management of kidney transplantation. Conventional nursing is limited to the care of hospitalized patients. It is difficult for patients to obtain professional nursing guidance after they are discharged from hospital, and the patients' self-management ability and medication compliance are poor, making it difficult to meet the needs of kidney transplant recipients for life-long medical treatment. Therefore, it is very important to carry out health education, improve medication compliance and control the occurrence of postoperative complications after discharge. Continuous nursing can provide professional care for discharged patients, improve their self-management ability, find abnormalities as early as possible, and get timely and effective treatment (6, 7). In this study, FTS nursing combined with continuous nursing of renal transplant patients was used to explore the influence of two nursing methods on self-care ability, medication compliance and quality of life of renal transplant patients.

## Data and Methods

### General Information

Sixty patients who received kidney transplantation in our hospital from January 2019 to January 2021 were randomly divided into the control group and the observation group with 30 patients in each group according to the random number table method. The control group was given FTS nursing, and the observation group was given continuous nursing on the basis of the control group.

#### Inclusion criteria

Age ≥18 years old; Those who meet the indications of kidney transplantation are treated; Chronic diseases, such as hypertension and diabetes, were stably controlled.

#### Exclusion criteria

Patients with multiple organ transplants; A second kidney transplantation; Patients with other serious diseases of organ foundation; Highly sensitive objects; Patients who failed to follow doctor's advice, withdrew from the study or had incomplete follow-up information. All patients and their families have obtained informed consent, and this study was approved by the hospital ethics committee.

### Research Methods

Patients in the control group received FTS nursing: The nursing management team established was mainly composed of researchers and doctors, nurses and dieticians in their hospitals studying the direction of renal transplantation. Specific qualification requirements are as follows: Medical personnel with bachelor degree or above and relevant certificates; More than 5 years working experience in kidney transplantation diagnosis and treatment; Good communication and coordination skills. ➀ Training and functions of nursing team: intensive training on nutrition therapy guidelines and related knowledge of kidney diseases for participants for one week in the form of lectures, group discussions, scene drills, etc. The head nurse of the department is responsible for coordinating the cooperation between the team members, the attending physician is responsible for the formulation of nutrition plan for patients, management and protection department is responsible for the formulation of nutrition plan, and the nutritionist is responsible for the modification. ➁ Personalized care plan: establish nutrition file for each patient, including general information of the patient (age, gender, educational level, marriage relationship, etc.), nutrition instruction list, biochemical test list, and physical test list. Body mass index (BMI) and total calories were calculated based on the patient's height and weight (carbohydrates accounted for 55–65% of the total daily calories, protein supply was 1.0–1.2g/kg per day, and fat supply accounted for about 25–35% of the total energy). Ensure the balance of water and electrolyte according to the biochemical test results, and adjust it according to patients' edema, blood pressure and electrolyte. ➂ Preoperative education: patients determine the operation time according to the operation sequence, and use oral education and written materials to education patients before operation, explain the theory and technology of kidney transplantation, preoperative preparation, what problems will occur during operation, how to solve the problems of surgeons and nurses, how to cooperate with patients, and tell them to keep a good attitude and build confidence in overcoming disease. ➃ Intraoperative preparation: Adius the room temperature to 26–28°C 1 h before the patient entered the operation room, and the room temperature was kept constant at 22–24°C until the end of the operation after the operation began, in order to reduce the heat loss of patients and provide a comfortable operation environment. In the operation and nursing operation, try to reduce the exposure of patients' bodies, reduce the overflow of flushing fluid during operation and keep the operating bed dry. The electronic heating infusion set was used to heat the infusion to 37°C, and the non-surgical area was covered with clothing or surgical towels with good thermal insulation performance to isolate it from the surrounding cold air, and to keep warm during the operation of the patient. Drugs with short half-life should be used as anesthetics to reduce the dosage of opioids. ➄ Postoperative observation: closely observe the changes of patients' condition in order to maintain the volume balance and water-electrolyte balance of patients with polyuria; In order to prevent rejection, immunosuppressants were used in time and correctly. If complications such as delayed recovery of transplanted renal function occur after operation, the responsible team shall follow up in time and take effective treatment and nursing measures, and arrange hemodialysis treatment if necessary to promote the recovery of renal function. ➅ Guidance at discharge: after the patient's condition is stable and reaches the discharge indications, give health education to the patients before discharge, know that the patient is taking medicine on time according to the doctor's advice, and check it regularly.

The observation group was additionally provided with continuous nursing on the basis of the control group. ➀ Follow-up after discharge: after the renal transplant patients are discharged from hospital, the responsible nurses are still responsible for continuous follow-up, tracking and guidance. Educate patients to pay attention to rest, combining rest with rest. Keep the living environment clean, quickly prevent colds and infection symptoms, and seek medical treatment. The necessity of long-term use of immunosuppressants was emphasized, and patients were urged to take medicine on time and pay attention to the complications of immunosuppressants. In diet, you should eat more foods rich in vitamins, take physiological doses of calcium for a long time, limit drinking water, and limit the intake of salt, protein and fat. ➁ Establish patient files: set up follow-up archives at the time of discharge, regular follow-up by phone and outpatient service, including the patient's vital signs, urine volume, rejection, diet, exercise status, drug use, etc., to master the patient taking immunosuppressive agents. ➂ Self-management education: in the follow-up nursing, guide the patients' self-care ability, and take medicine on time and according doctor's orders. The therapeutic effect of drugs, possible adverse reactions of drugs and precautions were explained to patients, so that they were psychologically prepared to observe whether accelerated rejection occurred. Strictly avoid accidents or missions, especially telling patients that increasing or decreasing the dose of immunosuppressants at will may cause serious consequences. At the same time, explain the purpose and requirements of each examinations are to the patient and emphasize the importance of regular review. ➃ Personalized adjustment of nursing plan: when the patients returned to our hospital for reexamination and telephone follow-up, we could show the patients that the operation had achieved the expected goal with objective and real examination data, thus further strengthening the patients' confidence in treatment. Patients who failed to achieve the expected goals were analyzed and discussed with the patients and their families, and more detailed care intervention plan was formulated. ➄ Psychological care: Pay attention to the patients' psychological status, conduct home care and give patients more social support. Ask the family members to pay attention to the emotional and social communication needs of patients. Hospitals regularly organize activities such as friendship and mutual assistance among patients, and provide more social support to patients through various channels.

### Observation Indicators

General data such as gender, age, BMI, educational level, marriage relationship, and dialysis method were collected and compared between the two groups. The postoperative indexes such as the time of first intake, postoperative blood glucose, serum creatinine, urea nitrogen, blood potassium, and postoperative hospital stay were recorded in the two groups. The comfort level of two groups after kidney transplantation was assessed using the Comfort Scale for Renal Transplant Recipients, which included four dimensions such as physiology, mental psychology, society and environment, with a total of 25 items. The items were scored according to the Likert 4-level scoring method with 1–4 points, the total score ranged from 25 to 100. A score of ≥62.5 indicates good comfort. A higher score indicated higher comfort, and the total score <62.5 indicated poorer comfort.

After a follow-up of six months, the self-care ability of the two groups was assessed using the self-management behavior scale, which included three dimensions (15 items), namely, cognitive symptom management practice, exercise, and communication with doctors, with a 5-level score of 1–5 points for each item, indicating complete non-compliance to complete compliance, 15–75 points, and the self-care ability of the patients was gradually enhanced with the improvement of the score. At the same time, according to the medication evaluation criteria, the medication compliance of two groups of patients was evaluated, which was divided into complete, partial and complete non-compliance. The self-rating scale for quality of life assessment was selected for assessment, including 4 dimensions of physical health, psychology/spirit, society/economy, and family. The score for each dimension was 0–30 points. A higher score indicated a better quality of life. Follow-up for 6 months, recording the hospitalization of patients due to complications.

### Statistical Methods

SPSS 20.0 software was used for processing. The measurement data of the experimental data were expressed as mean ± standard deviation, the *t* test was used for pairwise comparison. The count data were expressed as (rate) and the comparison was performed using chi-square test. The test level was α = 0.05, and *P* < 0.05 indicated that the difference was statistically significant.

## Results

### Comparison of General Information of Patients Between the Two Groups

There was no significant difference in general information such as gender, age, BMI, educational level, marriage relationship, and dialysis method between the two groups (*P*>0.05). As show in Table 1.

**Table 1:** Comparison of general information of patients between the two groups.

**Group**	**Age (years)**	**Gender**		**BMI(kg/m^**2**^)**	**Degree of education**	
		**Man**	**Woman**		**Junior secondary and below**	**Junior high school and above**
Control group (*n* = 30)	32.59 ± 6.17	21	9	23.13 ± 1.35	10	20
Observation group (*n* = 30)	31.85 ± 5.63	23	7	22.76 ± 1.26	12	18
*t*/*χ^2^* value	0.485	0.341		1.097	0.287	
*P* value	0.629	0.559		0.277	0.592	
**Group**	**Marital relations**			**Dialysis mode**		
	**Married**		**Be unmarried**	**Hemodialysis**		**Peritoneal dialysis**
Control group (*n* = 30)	21		9	24		6
Observation group (*n* = 30)	23		7	27		3
*χ^2^* value	0.341			1.176		
*P* value	0.559			0.278		

### Comparison of Postoperative Observation Indexes Between the Two Groups

**Figure 1:**
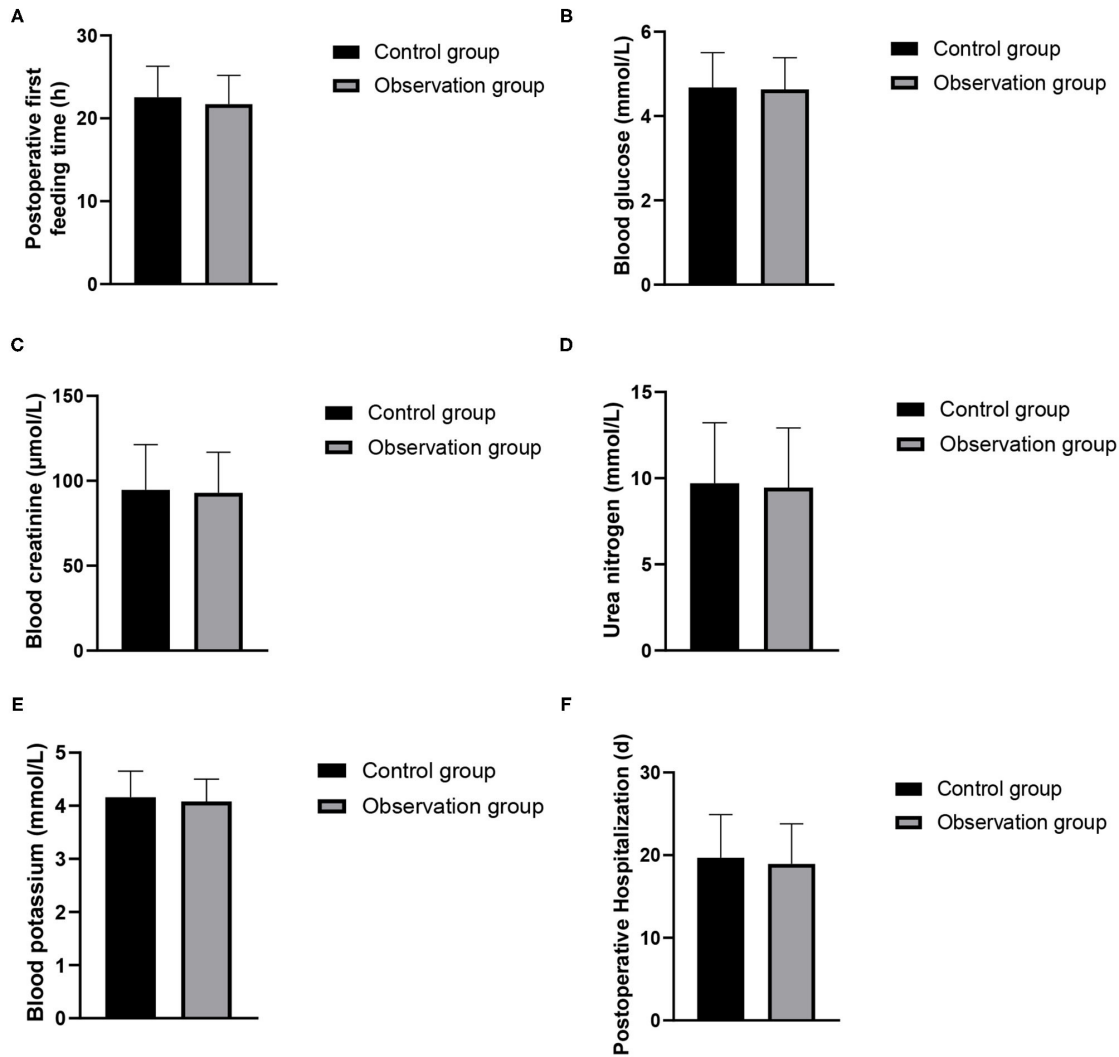
Postoperative observation indexes between the two groups. **(A)** Postoperative first feeding time; **(B)** blood glucose; **(C)** blood creatinine; **(D)** urea nitrogen; **(E)** blood potassium; **(F)** postoperative hospitalization.

There was no significant difference in the time of first intake, blood glucose, creatinine, urea nitrogen, blood potassium or postoperative hospital stay between the two groups (*P* > 0.05). As show in Figure 1.

### Comparison of Postoperative Comfort Indicators Between the Two Groups

**Figure 2:**
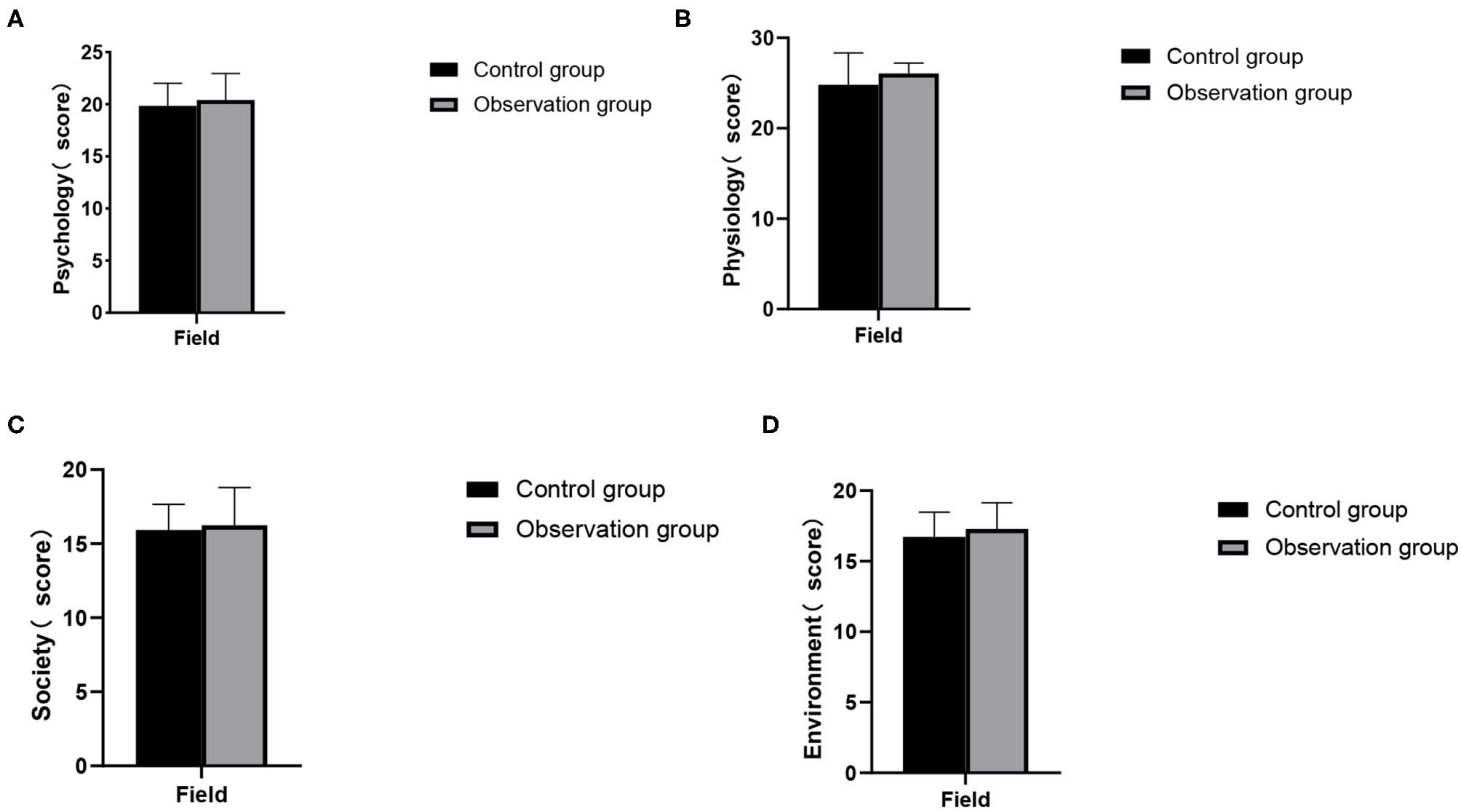
Postoperative comfort indicators between the two groups. **(A)** Psychology; **(B)** physiology; **(C)** society; **(D)** environment.

There was no significant difference in the postoperative physical, mental, psychological, social and environmental dimensions between the two groups (*P* > 0.05). As show in Figure 2.

### Comparison of Changes in Self-Care Ability Between the Two Groups

**Figure 3:**
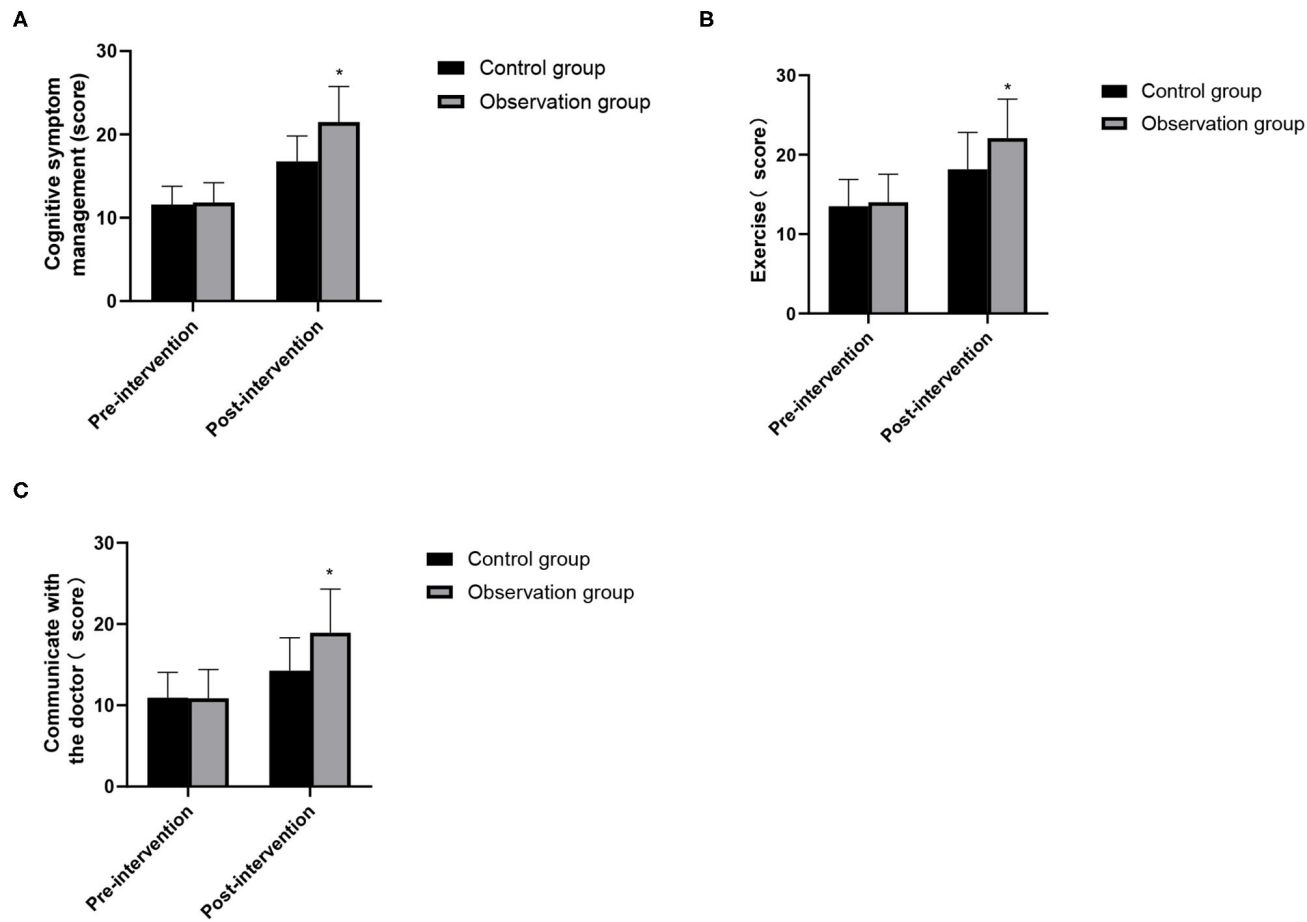
Changes in self-care ability between the two groups. **(A)** Cognitive symptom management; **(B)** exercise; **(C)** communicate with the doctor. Compared with the control group, *P < 0.05.

The scores of cognitive symptom management, exercise and communication with doctors in the two groups in pre-intervention were higher than those in pre-intervention, and the differences were statistically significant (*P* < 0.05). There was no significant difference between the two groups in pre-intervention (*P* > 0.05). In post-intervention, the scores of the observation group were higher than that of the control group and the differences were statistically significant (*P* < 0.05). As show in Figure 3.

### Comparison of Medication Compliance Between the Two Groups

**Figure 4:**
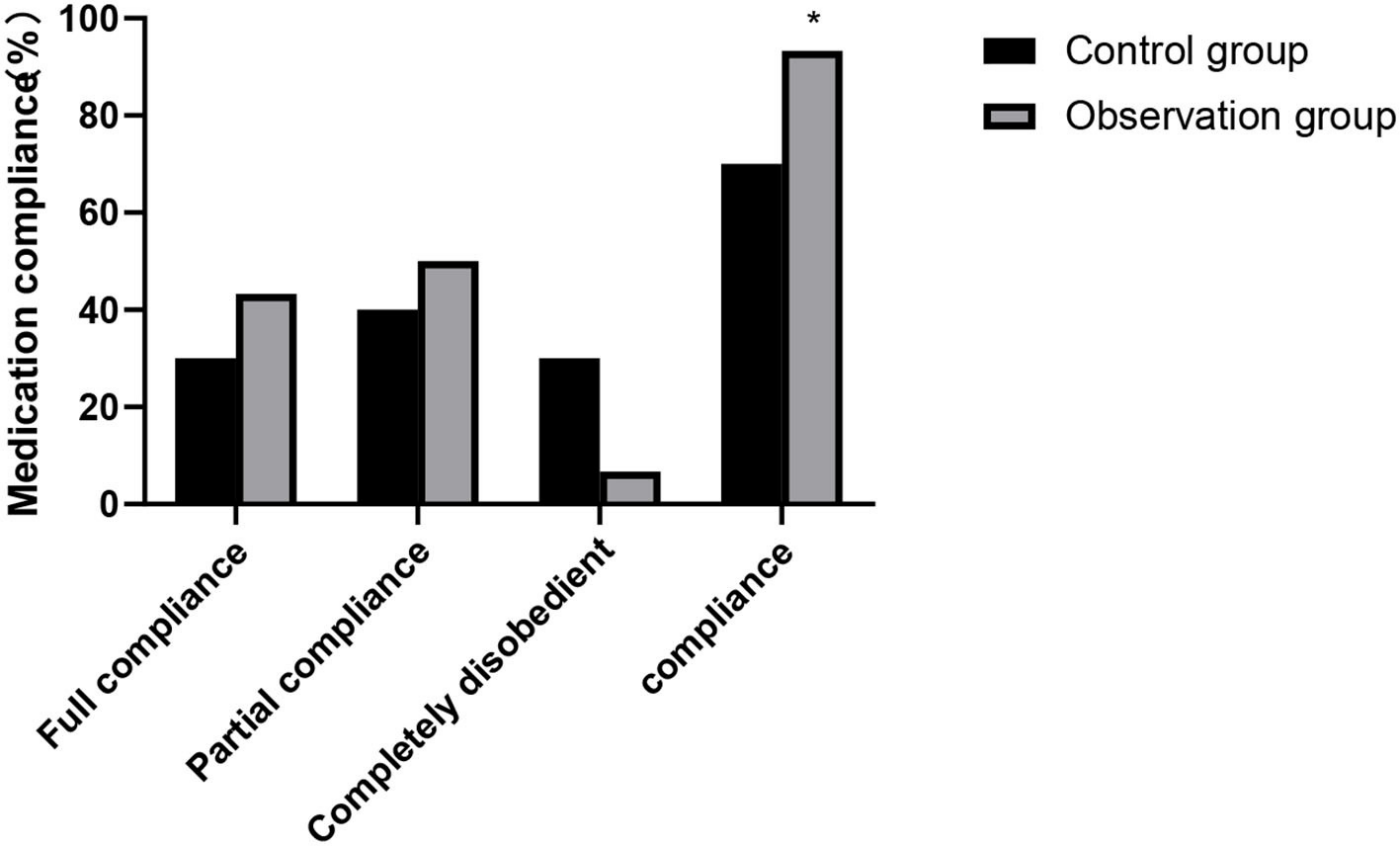
Medication compliance between the two groups. Compared with the control group, *P < 0.05.

The medication compliance of the observation group (93.33%) was higher than that of the control group (70.00%), and the difference was statistically significant (χ^2^ = 5.455, *P* = 0.020). As show in Figure 4.

### Comparison of Changes in Quality of Life Between the Two Groups

**Figure 5:**
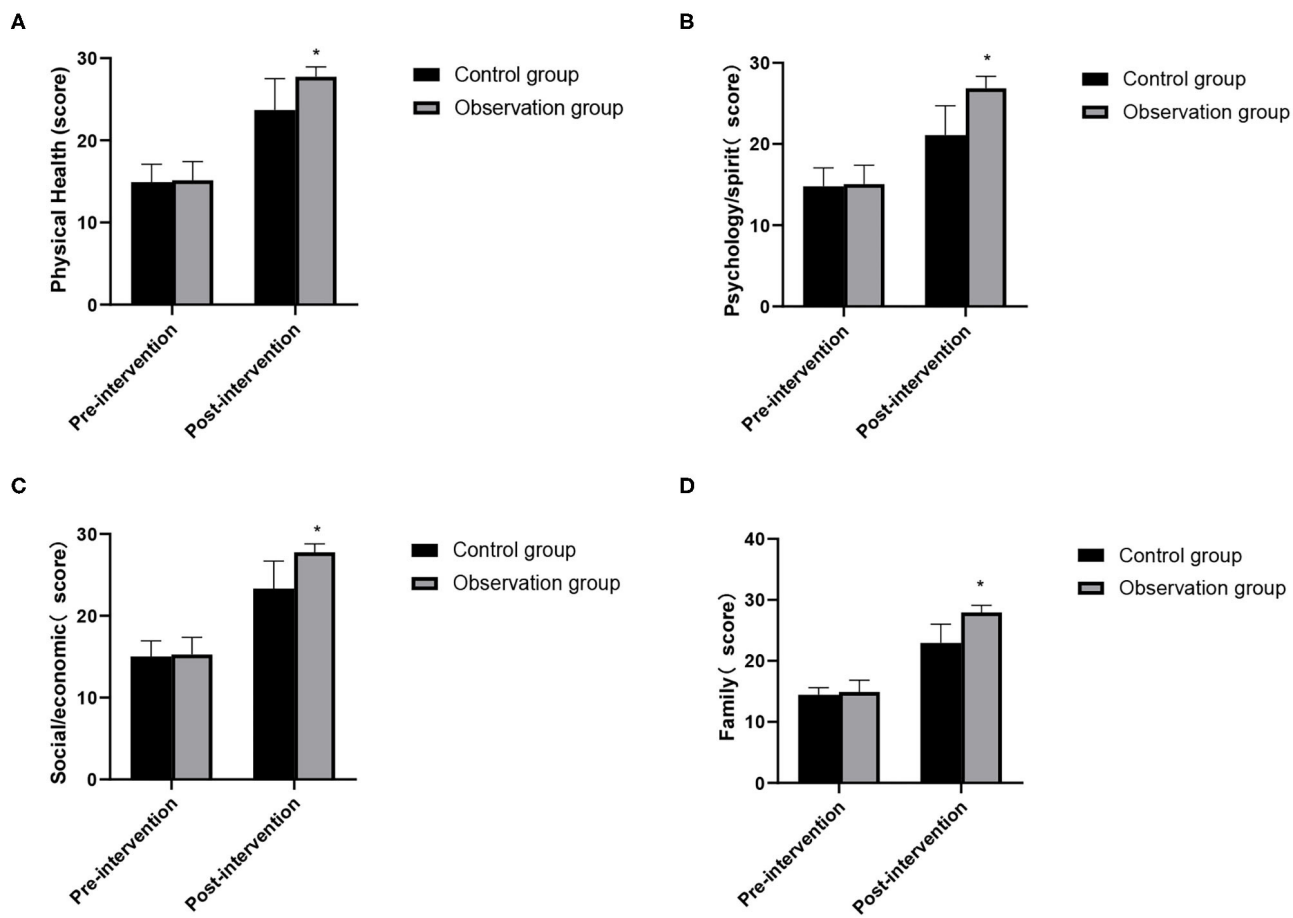
Changes in quality of life between the two groups. **(A)** Physical health; **(B)** psychology/spirit; **(C)** social/economic; **(D)** family. Compared with the control group, *P < 0.05.

In pre-intervention, there was no significant difference in the scores of physical health, psychology/spirit, society/economy, family and other dimensions of quality of life between the two groups (*P* > 0.05). In post-intervention, the scores of quality of life of the observation group were higher than those of the control group, and the differences were statistically significant (*P* < 0.05). As show in Figure 5.

### Hospitalization of Patients With Complications in Two Groups

**Figure 6:**
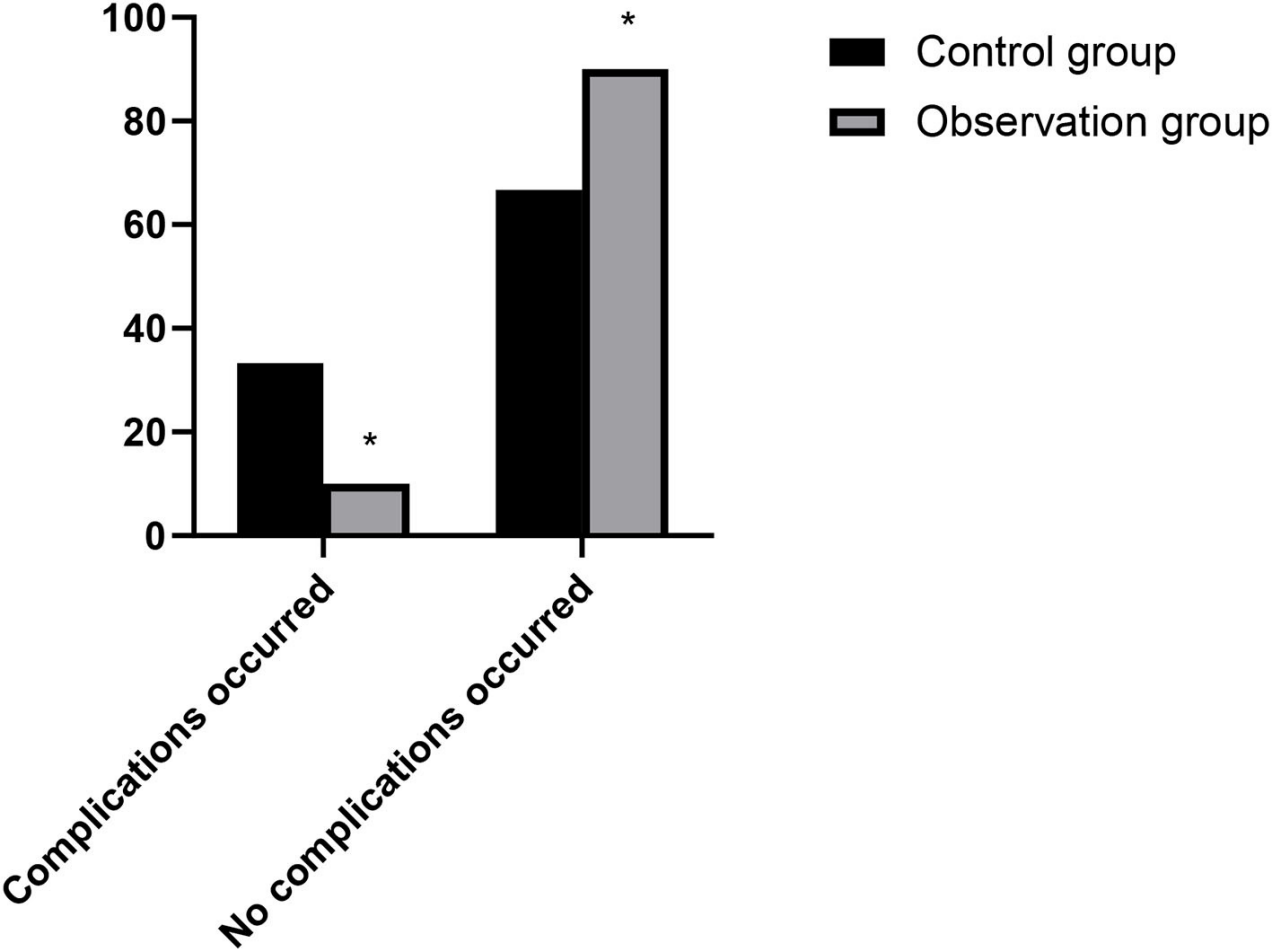
Hospitalization of patients with complications in two groups. Compared with the control group, *P < 0.05.

The complication admission rate of the observation group (10.00%) was lower than that of the control group (30.00%), and the differences were statistically significant (χ^2^ = 3.750, *P* = 0.035). As show in Figure 6.

## Discussion

The treatment of kidney transplantation is a kind of intense psychological stress for patients, which indirectly has adverse effects on patients' physiological and psychological function by affecting neuroendocrine and immune system. Therefore, it is of great significance to provide personalized and high-quality nursing care during the perioperative period to improve patients' tolerance to surgery (8). FTS nursing aims at blocking or reduce surgical stress reaction, reduce postoperative complications, accelerating patients' recovery and reduce hospital stay by optimizing multi-mode perioperative path and taking effective measures verified by evidence-based medicine (9, 10). Nursing continuously promotes the transformation of hospitals from “simple treatment mode” to “integrated service” (prevention service, treatment service, nursing service, rehabilitation and health care service). It can not only meet the needs of patients for health education, but also strengthen the enthusiasm of patients for rehabilitation, which is crucial for kidney transplant patients to improve the quality of life and return to normal social life (11, 12).

In this study, the FST nursing mode was adopted to comprehensively train medical staff on the diagnosis and nursing problems that may occur before, during and after the operation, standardize the diagnosis and nursing procedures of kidney transplantation, and strengthen the care and respect for the patients' humanity during the operation, so that patients can get the highest level of medical treatment and care during the perioperative period. The results showed that there was no significant difference in the time of first intake, postoperative blood glucose, creatinine, urea nitrogen, blood potassium and postoperative hospital stay between the two groups, and the overall postoperative comfort was higher. FST nursing model carries out nutritional assessment and screening before operation to reduce the risk of operation. Nurses should establish a good psychological defense mechanism for patients, and conduct personalized psychological intervention to help patients reduce their negative psychology (13). At the same time, we should strengthen the patient's knowledge education about kidney transplantation and its complications, improve the psychological resilience level of kidney transplant patients, relieve their psychological pressure and improve their mental health (14). Maintaining normal body temperature during operation can reduce the pressure of operation, the risk of organ dysfunction and the discomfort of patients. Choose short-acting anesthetics to reduce the adverse reactions of anesthetics, so that patients can begin to recover faster after operation. The patients' condition after transplantation should be closely observed to ensure their safety so that they can receive better care (15, 16).

After kidney transplantation, it is necessary to maintain the normal function of the transplanted kidney for a long time, and the recipient needs to take immunosuppressive drugs for life. Among them, the patient's non-compliance with drug treatment plan is the main cause of rejection and death of the transplanted kidney (17). Therefore, how to improve medication compliance of renal transplant patients is an urgent problem to be solved. Continuous nursing can strengthen the medical staff's observation of patients' medication compliance, educate patients' medication, and guide their medication behavior, thus greatly improving patients' medication compliance and understanding of medication plan (18). In this study, the sum of self-care ability scores such as cognitive symptom management, exercise and communication with doctors after continuous nursing intervention in two groups was higher than that in pre-intervention, and the scores in the observation group in post-intervention were higher than those in the control group. Medication compliance of the observation group was also better than that of the control group. It shows that active and continuous nursing could significantly improve patients' medication compliance and self-care ability, promote the survival and functional maintenance of transplanted kidney, and is also of great significance to improve patients' life quality (19).

Conventional nursing mainly relies on patients' voluntary reexamination. With the shortening of time, patients' attention is mostly focused on whether the indicators are normal or not, and the uneven compliance and self-care ability will affect the treatment effect and disease outcome (20). The results showed that the patients in the observation group had significantly higher quality of life scores than those in the control group after rapid rehabilitation surgery nursing combined with continuous nursing intervention. Patients in the observation group were also less likely to be readmitted for complications. It fully explained that FST nursing combined with continuous nursing strengthened the intervention measures for renal function protection, actively carried out rehabilitation exercise and psychological counseling nursing for patients after kidney transplantation, found early complications in time, and improved the long-term quality of life of patients.

FST nursing can help kidney transplant patients to obtain more stable postoperative renal function and other indicators and comfort. On this basis, combined with continuous nursing can improve the patient's self-care ability and medication compliance, which is of great significance to improve the quality of life of patients.

## Data Availability Statement

The original contributions presented in the study are included in the article/supplementary material, further inquiries can be directed to the corresponding author.

## Ethics Statement

The studies involving human participants were reviewed and approved by the Ethics Committee of Beijing Chao-Yang Hospital, Capital Medical University. The patients/participants provided their written informed consent to participate in this study.

## Author Contributions

LS is responsible for the revision of the paper and the guidance of the research. QJ is responsible for the design of the research. LZ is responsible for the inclusion of cases. ZL is responsible for the evaluation of the results. WC is responsible for the statistical analysis of the data. All authors contributed to the article and approved the submitted version.

## Conflict of Interest

The authors declare that the research was conducted in the absence of any commercial or financial relationships that could be construed as a potential conflict of interest.

## Publisher's Note

All claims expressed in this article are solely those of the authors and do not necessarily represent those of their affiliated organizations, or those of the publisher, the editors and the reviewers. Any product that may be evaluated in this article, or claim that may be made by its manufacturer, is not guaranteed or endorsed by the publisher.
